# Discovery of Novel Enhancers of Isoniazid Toxicity in *Mycobacterium tuberculosis*

**DOI:** 10.3390/molecules23040825

**Published:** 2018-04-04

**Authors:** Fabian Lentz, Norbert Reiling, Ana Martins, Joseph Molnár, Andreas Hilgeroth

**Affiliations:** 1 Institute of Pharmacy, Martin-Luther-University Halle-Wittenberg, 06120 Halle, Germany; fabian.lentz@pharmazie.uni-halle.de; 2 Research Center of Borstel, Leibniz Lung Center, 23845 Borstel, Germany; nreiling@fz-borstel.de; 3 German Center for Infection Research (DZIF), Partner Site Hamburg-Lübeck-Borstel, 23845 Borstel, Germany; 4 Department of Medical Microbiology, University of Szeged, 6720 Szeged, Hungary; anasfmartins@gmail.com (A.M.); molnar.jozsef@med.u-szeged.hu (J.M.)

**Keywords:** antibacterial enhancing activity, synthesis, derivatives, structure-activity, lead structure

## Abstract

The number of effective first-line antibiotics for the treatment of *Mycobacterium tuberculosis* infection is strongly limited to a few drugs. Due to emerging resistance against those drugs, second- and third-line antibiotics have been established in therapy with certain problems and also increasing mycobacterial resistance. An alternative to such novel drugs or combined therapeutic regimes which may reduce resistance development is finding enhancers of mycobacterial drug effectiveness, especially enhancers that counteract causative resistance mechanisms. Such enhancers may reduce the extracellular drug efflux mediated by bacterial efflux pumps and thus enhance the intracellular drug toxicity. We developed novel 1,4-dihydropyridines (DHPs) as potential efflux pump inhibitors with some determined P-gp affinities. The influence on the antituberculotic drug toxicity has been investigated for three prominent antituberculotic drugs. Exclusive and selective toxicity enhancing effects have been detected for isoniazid (INH) which could be related to certain substituent effects of the 1,4-DHPs. So, structure-dependent activities have been found. Thus, promising enhancers could be identified and a suggested efflux pump inhibition is discussed.

## 1. Introduction

The most recent tuberculosis (Tb) report of the World Health Organization (WHO) states that TB is the ninth leading cause of death worldwide and the leading cause from a single infectious agent, ranking above HIV/AIDS. In 2016, there were an estimated 1.3 million TB deaths among HIV-negative people (down from 1.7 million in 2000) and additional 374,000 deaths among HIV-positive people. In addition, an estimated 10.4 million people fell ill with TB [1]. A major concern is the steady increase of resistant and multidrug-resistant *Mycobacterium tuberculosis* (Mtb) complex strains. A total of 490,000 cases of multidrug-resistant TB (MDR-TB) have been reported for 2016. Almost half (47%) of these cases were in India, China, and the Russian Federation [1].

Isoniazid (INH), rifampicin (RIF), ethambutol (EMB), and pyrazinamide (PZA) are considered as first-line anti-TB drugs and form the core of standard treatment regimens [2]. If a given Tb isolate is found susceptible to the used drug regime, EMB or PZA are usually discontinued after two months. The ongoing therapy concentrates on the use of INH and RIF, which documents them as most important antituberculotic drugs [3]. INH has the strongest early bactericidal action and significantly contributes to rapidly making patients non-infectious. RIF has been shown to act on tubercle bacilli even in the state of nonreplicating persistence (NRP) [3].

Resistance against at least INH and RIF, which represent the fundamental components of any regimen for the treatment of drug-susceptible Tb, has been defined using the acronym MDR-TB (multidrug-resistant tuberculosis) [3]. In such a case, second-line antibiotics are used in a combined regime of four or five drugs including fluoroquinolone and aminoglycoside compounds [3]. An MDR-TB isolate which in addition also shows resistance against a fluoroquinolone and an aminoglycoside is termed extensively drug resistant (XDR-Tb). Only very limited therapy options do currently exist for an effective treatment of patients infected with XDR-Tb strains.

Novel antituberculotic drugs in development are strongly limited in number and their usage with partly insufficient knowledge about their mode of action and their effectiveness will be critical [4]. The costs of the therapy with such novel drugs will be too high for the developing countries which are mainly affected by Tb [5]. Moreover, the use of the established PZA becomes more doubtful due to observed initial resistances in Mtb strains as monoresistance or polyresistance [6,7].

One strategy to strengthen the effectiveness of the used antituberculotic drugs and to additionally combat the resistance developments may be the use of enhancers of the antituberculotic drug effects which are counteracted by an extracellular drug efflux. Only very few established drugs have been investigated to increase the intracellular toxicity of antituberculotic drugs. All of those used drugs have pharmacological activities like phenothiazines, verapamil, or reserpine which make them toxic for the use as potential enhancers of the antituberculotic drug activity by the inhibition of the extracellular efflux [8]. For most of those drugs, inhibitory activities of the human P-glycoprotein (P-gp) have been reported in earlier studies. P-gp is a transmembrane efflux pump in mammalian cells which is found overexpressed in cancer cells to export various anticancer drugs out of the cells, mostly independent of the compound structure [9]. Thus, P-gp mainly contributes to the MDR phenomenon in cancer treatment.

We developed a novel class of 1,4-dihydropyridines (DHP) with substituted phenyl residues both at the 4- and the nitrogen position of the DHP scaffold and investigated the P-gp inhibitory activities in a P-gp overexpressing cell assay system. The determined activities encouraged us to characterize potential compound effects on the toxicity of antituberculotic drugs in *Mycobacterium tuberculosis*. However, the compounds’ P-gp activities cannot be related to any activity data determined in Mtb because P-gp as an efflux pump is not present in Mtb. The novel compound class could be profiled in the use of the three most prominent antituberculotic drugs INH, RIF, and EMB with exclusive and selective effects on the INH toxicity which is discussed in relation to substituent effects.

## 2. Results and Discussion

### 2.1. Synthesis of the 1,4-Dihydropyridines

**Scheme 1:**
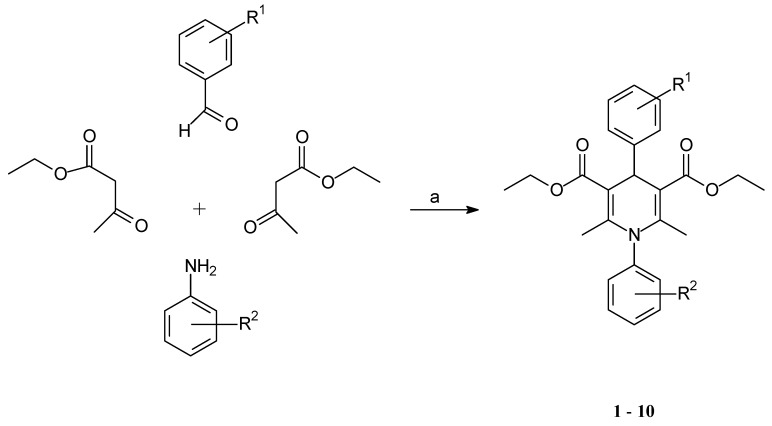
Synthesis of the 1,4-dihydropyridines **1**–**10**, (**a**) MeOH, EtOH, 8–10 h reflux.

For the synthesis of our 1,4-dihydropyridines **1**–**10** (Scheme 1) two equivalents of ethyl acetoacetate, one equivalent of the respective aromatic aldehyde, and one equivalent of the substituted aniline compound were heated under reflux conditions in alcohol, either methanol or ethanol. The reaction was monitored by TLC until no more of the starting compounds aromatic aldehyde or aniline derivatives were detectable. After removal of the solvent, the remaining oil was purified by column chromatography using a mixture of cyclohexane and ethyl acetate in a ratio of 75:25. From the unified compound-containing fractions, the target compounds were crystallized from methanol or ethanol or, alternatively, from a mixture of diethyl ether and methanol in a ratio of 1:5. The isolated compound yields varied from merely 2% to 21%. The lower yields may be caused by a reduced nucleophilic activity of the aniline nitrogen compared to that of ammonia used for the synthesis of the clinically used 1,4-dihydropyridines without a nitrogen substituent and higher isolated yields varying from the reaction conditions [10,11]. The spectroscopic compound characteristics were the conjugated ester carbonyl vibrational bond at about 1690 cm^−1^ in the IR spectra and the shift of the 4-proton of the 1,4-dihydropyridine scaffold to about 5 ppm in the ^1^H NMR spectra. In compounds **2**, **3**, and **7** with a 2′-anilino substituent, we found a double set of proton signals in the compound spectra which could be dedicated to two forms of isomers A and B, one with the 2-substituent at the *N*-phenyl residue on the left and the other with that substituent on the right site of the molecule due to a sterically hindered rotability of the nitrogen aniline bond. That hindered rotabality is caused by the close location of the 2- and 6-methyl substituent, respectively, to the near 2′-anilino substituent. In the case of the 3′-anilino substituted compounds **4**, **5**, and **8**–**10**, we found one set of proton signals, accordingly. Also the unsubstituted aniline compounds **1** and **6** resulted in just one set of proton signals.

### 2.2. P-glycoprotein Inhibitory Activity of the 1,4-Dihydropyridines

The P-gp inhibitory activity of the compounds has been determined in a mouse T-lymphoma cell model of two cell lines, one of which lacked P-gp expression and the other one expressing human P-gp after retroviral gene transfection with the human MDR1 gene encoding for P-gp. The latter cell line was cultured under colchicine conditions to provide a stable P-gp expression in the tested cells. Rhodamine 123 has been used as fluorescent P-gp substrate and the uptake of the substrate was measured in both cell lines without and with the use of our potential P-gp inhibitors. The fluorescence uptake was measured by flow cytometry. A fluorescence ratio (FAR) value was determined from the fluorescence amounts determined in the P-gp expressing cell line and the non-expressing cell line under inhibitor application after the values were corrected by those of the non-inhibitor-treated cells. FAR values > 1.1 prove a P-gp inhibitory activity of our compounds because the efflux of the fluorescent substrate in the P-gp expressing cell line will be decreased under inhibitor application.

The *N*-(2-tolyl) substituent of compound **2** showed the lowest P-gp inhibitory activity with a FAR value of 1.45 (Table 1, right column). If the 2-methyl function moved to the 3-position of the *N*-phenyl residue the activity was almost unchanged with a FAR value of 1.57 for compound **4**. If replaced with a chloro substitution, the activities of both compounds **3** and **5** slightly increased to 2.59 for the *N*-(2-chlorophenyl) substitution and to 3.61 for the *N*-(3-chlorophenyl) substitution. If combined with a 3-methoxy function at the 4-phenyl residue of compounds **7** and **8** both the *N*-2- and the *N*-3-tolyl derivatives showed the lowest activities again, with FAR values of 1.53 and 1.48, respectively, similar to the 4-phenyl unsubstituted compounds **2** and **4**. A *N*-(3-chlorophenyl) substitution combined with that 3-methoxyphenyl function in compound **9** was also not favourable with a FAR value of 1.57. If the 3-methoxy function at the 4-phenyl residue was replaced with a nitro group the activity of the *N*-(3-tolyl) compound **10** moderately increased with a FAR value of 4.05.

It can be stated that overall our compounds showed some P-gp inhibitory activities almost similar to the used standard verapamil with a FAR value of 1.40. If compared to the clinically used 1,4-dihydropyridine nifedipine with a FAR value < 1.1, the novel compounds show some improved activities, whereas nifedipine was not active. Those clinically used 1,4-dihydropyridines are unsubstituted at the nitrogen atom. Such an unsubstituted nitrogen is reported as an essential feature for their use as a calcium antagonist [10]. So, our novel compounds with their *N*-phenyl substitution will show no calcium antagonistic activity, but a preferred efflux pump inhibition due to their *N*-aryl substitution.

### 2.3. Antituberculotic Drug Toxicity Enhancing Properties of the 1,4-Dihydropyridines

With the determined P-gp inhibitory activities of our compounds we decided to determine a potential effect on the extracellular efflux of antituberculotic drugs similar to the reported drugs with P-gp inhibitory activities which could not be further developed due to their own pharmacological properties and resulting severe side-effects.

We started our studies with the most prominent antituberculotic drug, INH, that is also used prophylactically in latently infected patients [12]. The bacterial growth of the Mtb strain H37Rv expressing the green fluorescence protein (GFP) was characterized. We measured the GFP fluorescence related to the bacterial growth under the use of subinhibitory concentrations of INH alone and INH combined with our 1,4-DHPs to determine a potential drug toxicity enhancing effect. We observed increasing effects on the INH toxicity after compound application. The resulting decreased growth data were related to the INH growth inhibition alone. The growth inhibition data have been calculated accordingly and are shown in Table 1 as percent-increased growth inhibition data.

The *N*-phenyl and 4-phenyl substituted derivative **1** resulted in an increased growth inhibition of 35% compared to the inhibition of the sole INH. The introduction of a 2-methyl function in the *N*-phenyl residue of compound **2** increased the growth inhibition to 49%. A 2-chloro function of the *N*-phenyl residue led to a further increase to 61% for compound **3**. If both *N*-phenyl functions move to the 3-position of the *N*-phenyl residue the growth inhibition further increased to 64% for the 3-tolyl residue in compound **4** and to 80% for the 3-chlorophenyl substituent in compound **5**.

If combined with a 3-methoxy function at the 4-phenyl residue, we found a lowered growth inhibition for the unsubstituted *N*-phenyl residue in compound **6** of just 12%. The corresponding *N*-(2-tolyl) compound **7** again showed an increased growth inhibition of 52%. If the methyl group moved to the 3-position of the *N*-phenyl residue the corresponding compound **8** showed an increased growth inhibition to 86% and the *N*-(3-chlorophenyl) compound **9** showed a growth inhibition of 79%, similar to the 4-phenyl substituted derivative **5**. If the 3-methoxy function of the 4-phenyl residue was replaced with a nitro function and combined with the most favourable 3-methyl function of the *N*-phenyl residue in compound **10**, the growth inhibition was slightly reduced to 76% compared to compound **8**. The results demonstrate that *N*-3-phenyl substituted inhibitors with a 3-methoxy function at the 4-phenyl residue showed the best activities as enhancers of INH toxicity, as they increase the INH mediated growth inhibition up to 86% for compound **8**.

Next, we tested four of our best compounds identified in the growth inhibition assay with INH, namely compounds **8**, **5**, **10**, and **4**, to potentially enhance the growth inhibitory capacity of RIF under the same used conditions. However, only compound **10** slightly increased the antimycobacterial growth activity of RIF by 15%. All the other compounds showed almost unchanged rifampicin activity data. Finally, we investigated a potential inhibitory effect of our compounds on the growth inhibition of EMB in our assay system. We again used our four derivatives **8**, **5**, **10**, and **4**. We observed practically no effects on the growth inhibition of EMB under compound application with calculated data of 5% for compound **8**, 9% for compound **5**, 8% for compound **10**, and finally 2% for compound **4**.

Taken together, our data show that our novel compound class selectively enhances the antituberculotic activity of INH if compared to the other studied antituberculotic drugs. Those compounds with a 3-substitution of the *N*-phenyl residue preferably combined with a substituent at the 3-position of the 4-phenyl residue have been identified as lead compounds. Although the fact that a combination of our compounds with RIF and EMB showed no changes in the antituberculotic drug activity already suggested that our compounds themselves show no antituberculotic drug activity, we tested all compounds alone to inhibit the antituberculotic growth. None of the compounds showed any relevant activities, as expected. So, possible toxic effects of the compounds themselves can be excluded because such effects would reduce the growth of Mtb.

### 2.4. Efflux Pumps as a Potential Target of the 1,4-Dihydropyridines

In Mtb, efflux pumps of three families have been described; the major facilitator superfamily (MFS), the ATP-binding cassette (ABC) superfamily, and the resistance nodulation division family (RND) [8]. Several efflux pumps of these families have been related to antituberculotic drug resistance. All efflux pumps reported show no specificity for one single antituberculotic drug under normal cell culture conditions, rather they transport various drugs of the established first-line therapeutics [8].

As we observed a specific drug toxicity enhancing effect for INH when combined with our novel compounds, we wondered whether the used subinhibitory concentration of INH might have induced an efflux pump sensitive to our compounds as potential inhibitors. In a recent study, induction of efflux pumps was described for subinhibitory concentrations of the first-line antituberculotic drugs INH, RIF, and EMB [13].

For INH, the induction of the efflux pump encoding genes Rv1273c, Rv1819c, and Rv1250 has been described under conditions closely related to our cell culture conditions. Similarly, for RIF, induction of the Rv1273c and the Rv1819c genes has been described under the used subinhibitory conditions. For the used subinhibitory concentrations of EMB, efflux pump gene inductions have been observed for Rv1273c and Rv0194. As our compounds showed only effects on INH toxicity, but not on the RIF and EMB toxicity, we wondered whether Rv1250 might be the efflux pump which was selectively affected by our novel compounds. As no specific detectable substrates of one single efflux pump exist, it will be not possible to prove such a specify towards the respective efflux pump. However, it may be investigated in future studies.

## 3. Material and Methods

### 3.1. Chemical Reagents and Instruments

Commercial reagents were used without further purification. The ^1^H-NMR spectra (400 MHz) were measured using tetramethylsilane as internal standard. TLC was performed on E. Merck 5554 silica gel plates. The high-resolution mass spectra were recorded on a Finnigan LCQ Classic mass spectrometer.

### 3.2. General Procedure for the Synthesis of Compounds ***1***–***10***

One equivalent of the aniline compound and the aromatic aldehyde and two equivalents of ethyl acetoacetate were heated in a small volume of methanol or ethanol under reflux conditions. The reaction proceeding was followed by thin layer chromatography until no more starting compounds were detectable. Then, the solution was evaporated to dryness and the remaining oil was purified by column chromatography using silica gel and an eluent mixture of cyclohexane and ethyl acetate (3:1). The received compound-containing fractions were unified and evaporated. The compounds were finally crystallized either from the methanol or ethanol or from mixtures of diethyl ether and methanol in a ratio of 1: 5.

Diethyl 2,6-Dimethyl-1,4-diphenyl-1,4-dihydropyridine-3,5-dicarboxylate (**1**). Yield 16%; mp 153–157 °C; IR (ATR) ν = 1684 (C=O), 1198 (C–O–C) cm^−1^; ^1^H NMR (DMSO-*d_6_*) δ = 7.53–7.44 (m, 3H, 3-, 4-, 5-H of *N*-phenyl), 7.32–7.26 (m, 6H, 2-, 6-H of *N*-phenyl and 2-, 3-, 5-, 6-H of 4-phenyl), 7.19–7.14 (m, 1H, 4-H of 4-phenyl), 5.02 (s, 1H, 4-H), 4.08–4.01 (m, 4H, COOC**H**_2_CH_3_), 1.95 (s, 6H, C-2, -6-CH_3_), 1.16–1.12 (m, 6H, COOCH_2_C**H**_3_); *m*/*z* (ESI) 406.63 (M + H^+^).

Diethyl 2,6-Dimethyl-4-phenyl-1-(*o*-tolyl)-1,4-dihydropyridine-3,5-dicarboxylate (**2**). Yield 10%; mp 90–91 °C; IR (ATR) ν = 1691 (C=O), 1212 (C–O–C) cm^−1^; ^1^H NMR (DMSO-*d_6_*) δ = 7.44–7.11 (m, 18H, aromatic H of isomer A and B), 5.11 and 5.01 (2 x s, 2H, 4-H of isomer A and B), 4.08–3.98 (m, 8H, COOC**H**_2_CH_3_ of isomer A and B), 2.15 and 1.93 (2 x s, 6H, 2-CH_3_ of *N*-phenyl of isomer A and B), 1.88 (s, 12H, C-2-, -6-CH_3_ of isomer A and B), 1.14–1.11 (m, 12H, COOCH_2_C**H**_3_ of isomer A and B); *m*/*z* (ESI) 420.73 (M + H^+^).

Diethyl 1-(2-Chlorophenyl)-2,6-Dimethyl-4-phenyl-1,4-dihydropyridine-3,5-dicarboxylate (**3**). Yield 2%; mp 87–89 °C; IR (ATR) ν = 1693 (C=O), 1203 (C–O–C) cm^−1^; ^1^H NMR (DMSO-*d_6_*) δ = 7.74–7.35 (m, 10H, aromatic H of isomer A or B), 7.31–7.11 (m, 8H, aromatic H of isomer A or B), 5.04 and 4.99 (2 x s, 2H, 4-H of isomer A and B), 4.05–3.96 (m, 8H, COOC**H**_2_CH_3_ of isomer A and B), 1.93 and 1.87 (2 x s, 12H, C-2-, -6-CH_3_ of isomer A and B), 1.14–1.08 (m, 12H, COOCH_2_C**H**_3_ of isomer A and B); *m*/*z* (ESI) 440.71 (M + H^+^).

Diethyl 2,6-Dimethyl-4-phenyl-1-(*m*-tolyl)-1,4-dihydropyridine-3,5-dicarboxylate (**4**). Yield 8%; mp 99–103 °C; IR (ATR) ν = 1692 (C=O), 1205 (C–O–C) cm^−1^; ^1^H NMR (DMSO-*d_6_*) δ = 7.41–7.37 (m, 1H, 5-H of *N*-phenyl), 7.32–7.26 (m, 5H, 4-H of *N*-phenyl and 2-, 3-, 5-, 6-H of 4-phenyl), 7.19–7.14 (m, 1H, 4-H of 4-phenyl), 5.02 (s, 1H, 4-H), 4.07–4.01 (m, 4H, COOC**H**_2_CH_3_), 2.34 (s, 3H, 3-CH_3_- of *N*-phenyl), 1.96 (s, 6H, C-2-, -6-CH_3_), 1.16–1.12 (m, 6H, COOCH_2_C**H**_3_); *m*/*z* (ESI) 420.86 (M + H^+^).

Diethyl 1-(3-Chlorophenyl)-2,6-dimethyl-4-phenyl-1,4-dihydropyridine-3,5-dicarboxylate (**5**). Yield 3%; mp 124–125 °C; IR (ATR) ν = 1687 (C=O), 1201 (C–O–C) cm^−1^; ^1^H NMR (DMSO-*d_6_*) δ = 7.58–7.51 (m, 2H, 5-, 6-H of *N*-phenyl), 7.31–7.24 (s, 1H, 2-H of *N*-phenyl), 7.31–7.24 (m, 5H, 4-H of *N*-phenyl and 2-, 3-, 5-, 6-H of 4-phenyl), 7.18–7.14 (m, 1H, 4-H of 4-phenyl), 5.02 (s, 1H, 4-H), 4.07–4.02 (m, 4H, COOC**H**_2_CH_3_), 1.96 (s, 6H, C-2-, -6-CH_3_), 1.15–1.12 (m, 6H, COOCH_2_C**H**_3_); *m*/*z* (ESI) 440.71 (M + H^+^).

Diethyl 4-(3-Methoxyphenyl)-2,6-dimethyl-1-phenyl-1,4-dihydropyridine-3,5-dicarboxylate (**6**). Yield 2%; mp 116–117 °C; IR (ATR) ν = 1690 (C=O), 1199 (C–O–C) cm^−1^; ^1^H NMR (DMSO-*d_6_*) δ = 7.54–7.44 (m, 3H, 3-, 4-, 5-H of *N*-phenyl), 7.25–7.23 (m, 2H, 2-, 6-H of *N*-phenyl), 7.22 (t, J = 7.8 Hz, 1H, 5-H of 4-phenyl), 6.87 (dt, *J* = 7.8, 1.2 Hz, 1H, 6-H of 4-phenyl), 6.80–6.79 (m, 1H, 1H of 4-phenyl), 6.75 (ddd, *J* = 7.8, 2.6, 1.2 Hz, 1H, 4-H of 4-phenyl), 5.01 (s, 1H, 4-H), 4.11–4.00 (m, 4H, COOC**H**_2_CH_3_), 3.71 (s, 3H, CH_3_O), 1.95 (s, 6H, C-2, -6-CH_3_), 1.17–1.13 (m, 6H, COOCH_2_C**H**_3_); *m*/*z* (ESI) 436.64 (M + H^+^).

Diethyl 4-(3-Methoxyphenyl)-2,6-dimethyl-1-(*o*-tolyl)-1,4-dihydropyridine-3,5-dicarboxylate (**7**). Yield 10%; mp 98–99 °C; IR (ATR) ν = 1691 (C=O), 1211 (C–O–C) cm^−1^; ^1^H NMR (DMSO-*d_6_*) δ = 7.43–7.29 (m, 7H, aromatic H of *N*-phenyl of A and B), 7.24–7.20 and 7.19–7.15 (m, 2H, 5-H of 4-phenyl of A and B), 7.07–7.05 (m, 1H, aromatic H of *N*-phenyl of A or B), 6.89–6.87 and 6.84–6.82 (m, 2H, 6-H of 4-phenyl of A and B), 6.79–6.71 (m, 4H, 2-, 4-H of 4-phenyl of A and B), 5.09 and 4.99 (2 x s, 2H, 4-H of A and B), 4.08–3.99 (m, 8H, COOC**H**_2_CH_3_ of A and B), 3.72 and 3.69 (2 x s, 6H, CH_3_O of A and B), 2.15 and 1.97 (2 x s, 6H, 2-CH_3_ of *N*-phenyl of A and B), 1.88 and 1.87 (2 x s, 12H, C-2, -6-CH_3_ of A and B), 1.16–1.12 (m, 12H, COOCH_2_C**H**_3_ of A and B); *m*/*z* (ESI) 450.66 (M + H^+^).

Diethyl 4-(3-Methoxyphenyl)-2,6-dimethyl-1-(*m*-tolyl)-1,4-dihydropyridine-3,5-dicarboxylate (**8**). Yield 4%; mp 96–98 °C; IR (ATR) ν = 1691 (C=O), 1204 (C–O–C) cm^−1^; ^1^H NMR (DMSO-*d_6_*) δ = 7.41–7.37 (m, 1H, 5-H of *N*-phenyl), 7.29–7.26 (m, 1H, 4-H of *N*-phenyl), 7.22 (t, *J* = 7.9 Hz, 1H, 5-H of 4-phenyl), 7.04–7.03 (m, 2H, 2-, 6-H of *N*-phenyl), 6.86 (dt, *J* = 7.9, 1.2 Hz, 1H, 6-H of 4-phenyl), 6.79–6.78 (m, 1H, 2-H of 4-phenyl), 6.75 (ddd, *J* = 7.9, 2.6, 0.9 Hz, 1H, 4-H of 4-phenyl), 5.01 (s, 1H, 4-H), 4.09–4.02 (m, 4H, COOC**H**_2_CH_3_), 3.72 (s, 3H, CH_3_O), 2.33 (s, 3H, 3-CH_3_ of *N*-phenyl), 1.96 (s, 6H, C-2, -6-CH_3_), 1.17–1.14 (m, 6H, COOCH_2_C**H**_3_); *m*/*z* (ESI) 472.72 (M + Na^+^).

Diethyl 1-(3-Chlorophenyl)-4-(3-Methoxyphenyl)-2,6-dimethyl-1,4-dihydropyridine-3,5-dicar-boxylate (**9**). Yield 3%; mp 108–110 °C; IR (ATR) ν = 1692 (C=O), 1203 (C–O–C) cm^−1^; ^1^H NMR (DMSO-*d_6_*) δ = 7.59–7.54 (m, 2H, 5-, 6-H of *N*-phenyl), 7.42 (s, 1H, 2-H of *N*-phenyl), 7.25–7.24 (m, 1H, 4-H of *N*-phenyl), 7.23 (t, *J* = 7.8 Hz, 1H, 5-H of 4-phenyl), 6.87 (dt, *J* = 7.8, 1.2 Hz, 1H, 6-H of 4-phenyl), 6.78 (t, *J* = 1.2 Hz, 1H, 2-H of 4-phenyl), 6.77 (dt, *J* = 7.8 Hz, 1.2 Hz, 1H, 4-H of 4-phenyl), 5.02 (s, 1H, 4-H), 4.08 (A**B**X_3_, *J* = 10.9, 7.1 Hz, 2H, COOC**H**_A_ H_B_CH_3_), 4.07 (A**B**X_3_, *J* = 10.9, 7.0 Hz, 2H, COOCH_A_**H**_B_CH_3_), 3.73 (s, 3H, CH_3_O), 1.98 (s, 6H, C-2, -6-CH_3_), 1.16 (AB**X**_3_, *J* = 7.1, 7.0 Hz, 6H, COOCH_2_C**H**_3_); *m*/*z* (ESI) 492.89 (M + Na^+^). 

Diethyl 2,6-Dimethyl-4-(3-nitrophenyl)-1-(*m*-tolyl)-1,4-dihydropyridine-3,5-dicarboxylate (**10**). Yield 21%; mp 114–115 °C; IR (ATR) ν = 1694 (C=O), 1206 (C–O–C) cm^−1^; ^1^H NMR (DMSO-*d_6_*) δ = 8.16 (t, *J* = 2.0 Hz, 1H, 2-H of 4-phenyl), 8.07 (dt, *J* = 7.8, 2.0 Hz, 1H, 4-H of 4-phenyl), 7.76 (dt, *J* = 7.8, 2.0 Hz, 1H, 6-H of 4-phenyl), 7.63 (t, *J* = 7.8 Hz, 1H, 5-H of 4-phenyl), 7.44–7.40 (m, 1H, 5-H of *N*-phenyl), 7.32–7.29 (m, 1H, 4-H of *N*-phenyl), 7.17–7.14 (m, 2H, 2-, 6-H of *N*-phenyl), 5.09 (s, 1H, 4-H), 4.10–3.99 (m, 4H, COOC**H**_2_CH_3_), 2.36 (s, 3H, 3-CH_3_ of *N*-phenyl), 2.00 (s, 6H, C-2, -6-CH_3_), 1.16–1.13 (m, 6H, COOCH_2_C**H**_3_); *m*/*z* (ESI) 465.72 (M + H^+^).

### 3.3. P-gp Inhibitory Activity

Two cell lines, a mouse T-lymphoma parental cell line L5178Y and a P-gp expressing subline L5178Y mdr, which resulted from retrovirus-mediated gene transfection, were cultured at 37 °C under carbon dioxide containing atmosphere (5%) in McCoys (San Marcos, TX, USA) 5A medium containing 10% of fetal calf serum, l-glutamine (2 mM) and 5 mL of a gentamicin solution (5 mg/mL). The medium used for culturing the P-gp expressing subline L5178Y mdr has been additionally supplemented with colchicine (60 ng/mL) to ensure a stable P-gp expression.

Cells of the mouse T-lymphoma parental cell line and the P-gp expressing subline were taken in an adjusted concentration of 1 × 10^6^ cells per mL medium and resuspended in serum free McCoys 5A medium. 0.5 mL Aliquots were filled into Eppendorf (Hamburg, Germany) centrifuge tubes. The test compounds **1**–**10** taken from prepared stock solutions in dimethylsulfoxide (1.0 mg/mL) were added in a volume of 5 µL. After 10 min of inhibitor preincubation at rt the P-gp substrate rhodamine 123 was added using 5 µL of a 0.5 mM solution in water reaching a final inhibitor concentration of 1 µM. Incubation was continued for 40 min at 37 °C. After that, the cells were centrifuged and washed twice with phosphate-buffered saline (PBS). Then, they were resuspended in PBS for measurement. The non-inhibitor containing cells were treated in the same way as the inhibitor preincubated cells. The fluorescence uptake of rhodamine 123 within a number of 1 × 10^4^ counted cells was determined by flow cytometry using a Becton Dickinson FACScan (Franklin Lakes, NJ, USA) flow cytometer. The fluorescence activity ratio (FAR) value was calculated from the quotient of the determined fluorescence uptake ratios of the P-gp expressing cell (MDR) line and the non-P-gp expressing parental cell line (parental). Both ratios have been corrected by division with the fluorescence determined in the inhibitor untreated control cell lines following the equation for the *FAR*:

MDR treated/MDR control

Parental treated/Parental control

### 3.4. Mtb Growth Inhibition Assay

Green fluorescence-expressing Mtb H37Rv bacteria were employed for growth analysis as previously described [14]. In brief, 2 × 10^6^ bacteria were cultured in 7H9 medium supplemented with 10% oleic acid-albumin-dextrose-catalase (OADC), 0.05% Tween 80, and 0.2% glycerol in a total volume of 100 µL in a black 96-well plate with a clear bottom (Corning Inc., Corning, NY, USA) sealed with an air-permeable membrane (Porvair Sciences, Wrexham, UK). Preincubation was carried out for seven days with subinhibitory concentrations of INH (0.08 µg/mL), RIF (0.01 µg/mL), and EMB (3 µg/mL) as described [11]. Bacterial growth was measured under 1,4-DHP compound concentrations of 10 µg/mL in triplicates as relative light units at 528 nm after excitation at 485 nm in a fluorescence microplate reader after seven days of culture (Synergy 2, Biotek, Winooski, VT, USA). The fluorescence values determined for the combined antituberculotic drug and compound application were each related to those of the determined values for the antituberculotic drug activity alone that were set to 100%. So, reduced growth values have been given for the combination of INH and the used compounds. The growth inhibition has been calculated accordingly as the difference between the determined percent growth values and 100%.

## 4. Conclusions

Antituberculotic therapy is limited to a small number of drugs. Resistance against those drugs are critical due to a lack of alternatives. One promising strategy is to increase the cellular antituberculotic drug effectiveness, which may also decrease the risk of resistance development caused by efflux pump activity. We developed novel 1,4-dihydropyridines in a simple one-pot reaction that were demonstrated to selectively enhance INH toxicity in Mtb. The observed selectivity may be explained by efflux pump inhibition by the compounds under the efflux pump inducing conditions of subinhibitory concentrations of antituberculotic drugs. Such efflux pump inhibiting properties have not been reported for 1,4-dihydropyridines before. In the case of the clinically used 1,4-dihydropyridines, such effects are not expected because they show almost no effect towards P-gp, as demonstrated for nifedipine. Structure-dependent activities have been found and the first lead compounds have been identified, representing the first selective enhancers of antituberculotic drug activity.

## Figures and Tables

**Table 1:** Isoniazid (INH) toxicity enhancing activity and P-gp-inhibiting properties of target compounds **1**–**10**.

Cpd.	R^1^	R^2^	Increased Growth Inhibition (%) ^a,b^	FAR Value ^a^
**1**	H	H	35 ± 1.6	n.d. ^c^
**2**	H	2-Me	49 ± 0.7	1.45 ± 0.09
**3**	H	2-Cl	61 ± 6.3	2.59 ± 0.14
**4**	H	3-Me	64 ± 2.5	1.57 ± 0.09
**5**	H	3-Cl	80 ± 0.8	3.61 ± 0.22
**6**	3-MeO	H	12 ± 0.7	2.21 ± 0.31
**7**	3-MeO	2-Me	52 ± 1.0	1.53 ± 0.09
**8**	3-MeO	3-Me	86 ± 1.3	1.48 ± 0.08
**9**	3-MeO	3-Cl	79 ± 1.3	1.57 ± 0.09
**10**	3-NO_2_	3-Me	76 ± 1.3	4.05 ± 0.71

^a^ Mean of three determinations; ^b^ percent growth inhibition, each related to INH growth inhibition; ^c^ n.d., not determined.
